# Gastric artery embolization: studying the effects of catheter type and injection method on microsphere distributions within a benchtop arterial model

**DOI:** 10.1186/s12938-020-00794-z

**Published:** 2020-06-26

**Authors:** Shaphan R. Jernigan, Jason A. Osborne, Gregory D. Buckner

**Affiliations:** 1 Departments of Biomedical, Mechanical and Aerospace Engineering, North Carolina State University, Raleigh, NC 27695 USA; 2 Department of Statistics, North Carolina State University, Raleigh, NC 27695 USA

**Keywords:** Gastric artery, Embolization, Vessel targeting, Reflux

## Abstract

**Aims:**

The objective of the study is to investigate the effect of catheter type and injection method on microsphere distributions, specifically vessel targeting accuracy.

**Materials and methods:**

The study utilized three catheter types (a standard end-hole micro-catheter, a Surefire anti-reflux catheter, and an Endobar occlusion balloon catheter) and both manual and computer-controlled injection schemes. A closed-loop, dynamically pressurized surrogate arterial system was assembled to replicate arterial flow for bariatric embolization procedures. Four vessel branches immediately distal to the injection site were targeted for embolization. Embolic microspheres were injected into the model using these  three catheter types and both manual and computer-controlled injections.

**Results:**

Across all injection methods, the catheter effect on the proportion of microspheres to target vessels (vs. non-target vessels) was significant (*p* = 0.005). The catheter effect on the number of non-target vessels embolized was nearly significant (*p* = 0.059). Across all catheter types, the injection method effect was not statistically significant for either of two outcome measures (percent microspheres to target vessels: *p* = 0.265, number of non-target vessels embolized: *p* = 0.148).

**Conclusion:**

Catheter type had a significant effect on targeting accuracy across all injection methods. The Endobar catheter exhibited a higher targeting accuracy in pairwise comparisons with the other two injection catheters across all injection schemes and when considering the Endobar catheter with the manifold injection method vs. each of the catheters with the manual injection method; the differences were significant in three of four analyses. The injection method effect was not statistically significant across all catheter types and when considering the Endobar catheter/Endobar manifold combination vs. Endobar catheter injections with manual and pressure-replicated methods.

## Background

Bariatric embolization of the gastric arteries has recently emerged as a treatment option for human obesity [1–7]. Limited clinical trials, retrospective analyses, and animal trials have shown a reduction in body weight and/or body mass index (BMI) following embolization of these vessels. Weight loss is believed to be induced through reduced levels of ghrelin, an appetite-stimulating (orexigenic) hormone produced primarily by cells in the stomach fundus [1]. Kipshidze et al. [2] performed the first human trials specifically to study this phenomenon (*n* = 5) and noted post-procedural decreases in body weight in follow-up visits ranging from 1 month (average of 10% decrease) to 20–24 months (average of 17% decrease). Similarly, ghrelin levels were reduced below the baseline at 1-, 3-, 6-, and 12-month follow-up periods (29%, 36%, 19%, and 21% reductions, respectively). A retrospective human study [3] of patients being treated for gastrointestinal (GI) bleeding found a significant reduction in body weight 3 months post-procedure for patients undergoing left gastric artery embolization (7.3% reduction, *n* = 19) compared with a control group undergoing embolization of another artery (2% reduction, *n* = 28). Multiple animal trials, summarized by Anton et al. [1], have shown similar reductions in body weight and/or ghrelin levels with left gastric artery embolization.

**Fig. 1:**
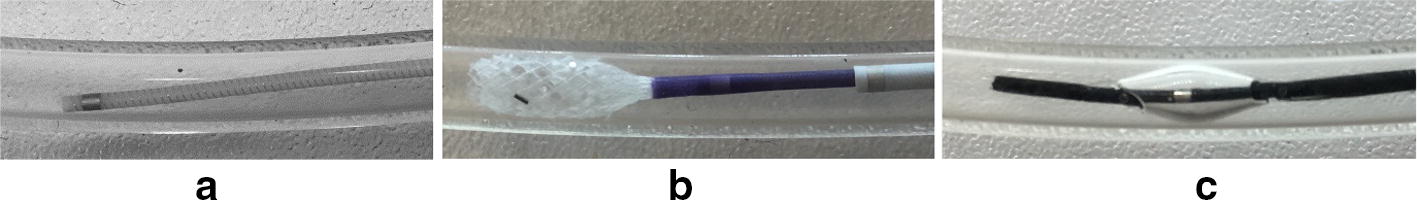
Catheters used for this study (magnified view of tips): **a** standard end-hole micro-catheter (Renegade HI-FLO, 0.027 in ID, 105 cm length), **b** Surefire anti-reflux catheter (0.027 in ID, 120 cm length) and **c** Endobar occlusion balloon catheter (0.022 in ID, 150 cm length)

**Fig. 2:**
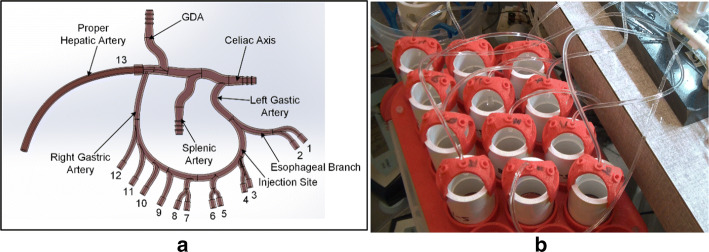
Benchtop arterial model: **a** 3D CAD illustration with vessel numbering and labels; **b** filters at the terminations of the esophageal and gastric arteries (labeled 1 through 12 in **a**)

Proper administration of embolics is essential for procedural efficacy and prevention of particle delivery to non-target vessels. This study seeks to assess the effects of injection catheter type and injection method on two primary outcomes: vessel targeting accuracy and the distribution of the microspheres among the target vessels. The study utilizes three catheter types (a standard end-hole micro-catheter, a Surefire anti-reflux catheter, and an Endobar occlusion balloon catheter) and both manual and computer-controlled injection schemes (Fig. 1). A benchtop arterial model replicates the hemodynamics (pressures, flow rates, pulsatile flow characteristics) and anatomical geometry (vessels diameters) both proximal and distal to the embolic injection point (Fig. 2). Catheter type is shown to have a significant effect on targeting accuracy across all injection methods.

## Results

A total of 21 tests (Table 1) were completed utilizing an end-hole catheter (*n* = 6), a Surefire anti-reflux catheter (*n* = 6), and an Endobar prototype (*n* = 9), thus providing two to three replicate runs of each factorial combination. Linear models with factorial effects were fit using SAS PROC GLM [8] so that analysis of variance could be used to test the significance of the observed effects of catheter type and injection method on two response variables: the proportion of microspheres depositing in target vessels and the number of non-target vessels embolized. These models included main effects for the factors catheter type and injection method and their interaction. These effects were all nested within another effect created for the unique design point using the Endobar catheter and the Endobar manifold injection method in a model appropriate to the augmented, complete, crossed, 3 × 3 + 1 factorial design. In addition, simple (pairwise) and complex (involving more than two combinations) contrasts among averages were carried out to further characterize the effects of catheter type and injection method.

**Table 1:** Data for each test conducted

Catheter	Injection method	Injection rate^a^ (mL/min)	Post-injection flow rates per vessel, vessels 1–12 (mL/min)	Number non-target vessels embolized	Percent microspheres to target vessels	Total microspheres counted
End-hole	Syringe pump	1.50	2.06	5	24.48%	192
	Syringe pump	1.50	1.45	6	52.73%	1117
	Pressure replicated	3.40	1.27	6	52.06%	559
	Pressure replicated	3.40	0.33	8	30.19%	2183
	Manual	8.29	0.39	7	38.60%	3927
	Manual	10.80	0.22	8	33.35%	3094
Surefire	Syringe pump	1.50	1.42	6	77.91%	2218
	Syringe pump	1.50	2.16	3	73.80%	1500
	Pressure replicated	3.40	2.04	4	62.10%	1103
	Pressure replicated	3.40	1.32	6	63.53%	2328
	Manual	10.75	1.43	6	47.31%	2579
	Manual	7.58	1.21	6	59.58%	3105
Endobar	Endobar manifold	1.50	3.63	0	96.30%	729
	Endobar manifold	1.50	3.57	0	94.36%	780
	Syringe pump	1.50	2.91	2	81.71%	257
	Syringe pump	1.50	1.23	0	99.43%	174
	Pressure replicated	3.40	2.04	4	85.06%	1486
	Pressure replicated	3.40	1.35	6	65.43%	2183
	Manual	5.25	0.26	8	37.98%	2383
	Manual	6.56	4.94	0	99.67%	2148
	Manual	5.62	1.85	5	67.96%	1910

^a^Manual injection rates were estimated from video recorded during each test

### Vessel targeting accuracy

**Fig. 3:**
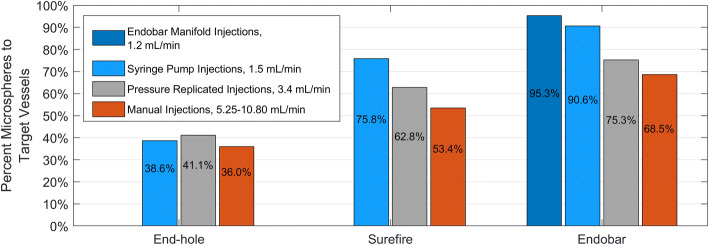
Vessel targeting accuracy (percent microspheres to target vessels) for each catheter type and injection method. Catheter effect was statistically significant (*p* = 0.005), whereas injection method effect was not statistically significant (*p* = 0.265) for this outcome measure. For the Endobar catheter, differences in outcomes between the Endobar manifold and non-automated injections were not statistically significant (*p* = 0.119)

**Fig. 4:**
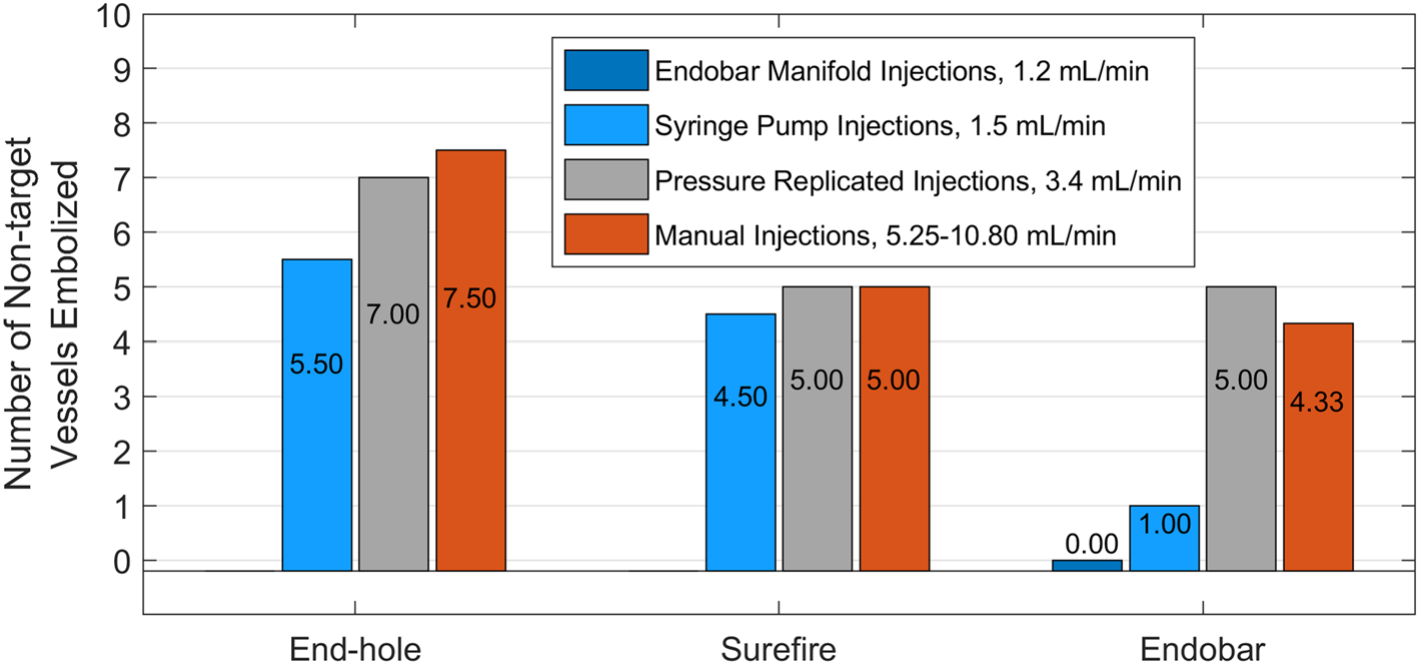
Non-target embolization (inaccuracy) for each catheter type and injection method. Catheter effect was nearly significant (*p* = 0.059), with a pairwise comparison of extremes (End-hole and Endobar) being highly significant (*p* =0.0032) and the ordering of outcome means (target and nontarget) across the three catheters being preserved. Injection method effect was not statistically significant across all catheter types (*p* = 0.1476); however, for the Endobar catheter, the difference in outcomes was significant in a pairwise comparison between the Endobar manifold and manual injections (*p* = 0.041)

Targeting accuracy was assessed using the percentages of microspheres deposited in the filters of target vessels (gastric arteries labeled 3 through 6 in Fig. 2) vs. non-target and the number of non-target vessels embolized. Across all injection methods, the catheter effect on the proportion of microspheres to target vessels (vs. non-target vessels) was significant (*p* = 0.005, Fig. 3). The catheter effect on the number of non-target vessels embolized was nearly significant (*p* = 0.059, Fig. 4). Pairwise statistical comparisons between catheters were also performed. Comparisons of the Endobar catheter/Endobar manifold combination vs. non-Endobar catheters with manual injections were also included, since manual injections are clinical standards for non-Endobar catheters.

When compared directly with the end-hole catheter, the Endobar catheter had significantly higher targeting accuracy (percent microspheres to target vessels) both across all injection methods (*p* < 0.001) and when considering the Endobar catheter/Endobar manifold combination vs. the end-hole catheter/manual injection combination (*p* = 0.004). Similarly, direct comparisons with the Surefire catheter revealed the Endobar catheter had a higher targeting accuracy across all injection methods (*p* = 0.059). This difference was more pronounced when considering the Endobar catheter/Endobar manifold combination vs. the Surefire catheter/manual injection combination (*p* = 0.028).

### Microsphere distributions

**Fig. 5:**
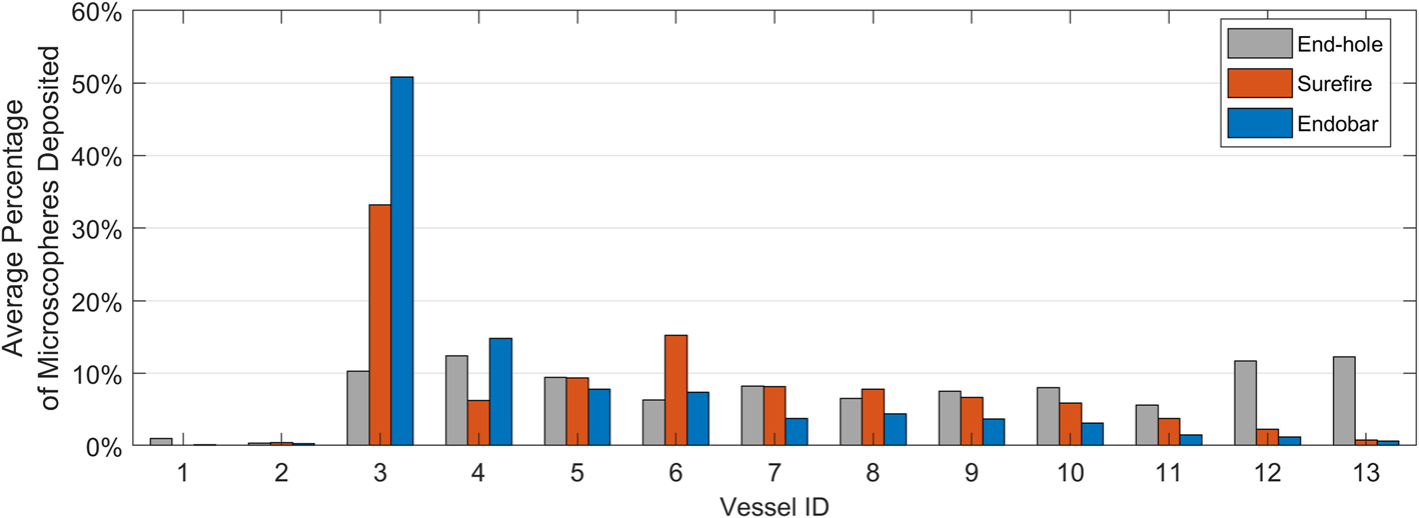
Distribution of microspheres among the target vessels, averaged for each factorial combination

Microsphere distributions for each test were computed by dividing the microsphere count per vessel by the quantity of microspheres collected in all filters (Fig. 5). Although they comprise 15.4% of all the vessels, on average less than 1% of the microspheres were deposited in the esophageal branches (Table 2) for all tests, indicating minimal reflux for all catheters and injection methods; zero microspheres were detected in the esophageal branches for 13 of the 21 tests. Microsphere deposition in the hepatic arteries was minimal (an average of less than 1% of microspheres per test) for the Surefire and Endobar catheters; however, an average of 12.29% of microspheres per test were deposited in the hepatic arteries for the end-hole catheter. With the Surefire and Endobar catheters, the microspheres preferentially deposited in vessels immediately downstream from the catheter tip; an average of 64.04% and 80.88% were deposited in vessels 3–6, while an average of 34.68% and 17.94% were deposited in vessels 7–12 for the Surefire and Endobar catheters, respectively (Table 2).

**Table 2:** Microsphere distributions for all tests

Catheter	Average proportion of microspheres to vessel group			
	Esophageal branch	Gastric arteries		Hepatic artery
	Vessels 1–2^b^	Vessels 3–6^a^	Vessels 7–12^b^	Vessel 13^b^
End-hole	1.40%	38.57%	47.73%	12.29%
Surefire	0.46%	64.04%	34.68%	0.82%
Endobar	0.49%	80.88%	17.94%	0.70%

^a^Target, ^b^Non-target

For the Surefire and Endobar catheters, targeting accuracy decreased with higher injection rates; injections with the lowest rates (1.5 mL/min) yielded the highest average targeting accuracies (Figs. 3 and 4). This trend was observed for the end-hole catheter but only in regard to number of non-target vessels embolized. In spite of these observed trends, the injection method effect was not statistically significant across all catheter types for either of two outcome measures (percent microspheres to target vessels: *p* = 0.265; number of non-target vessels embolized: *p* = 0.148). To assess the value of the Endobar manifold over non-automated methods, statistical comparisons were made between Endobar manifold injections and the average of manual and pressure-replicated injection methods. In regard to percent microspheres to target vessels, the manifold exhibited higher targeting accuracy compared with the average of the non-automated injection methods; however, the difference was not statistically significant (*p* = 0.119, Fig. 3). However, the difference in number of non-target vessels embolized was significant in a pairwise comparison between the Endobar manifold and manual injections (*p* = 0.041).

## Discussion

### Vessel targeting accuracy

The trend toward higher non-target deposition with increased injection rate is somewhat intuitive with the Endobar and Surefire catheters. Higher injection rates naturally tend to propel microspheres further toward the RGA and into the hepatic arterial branch. Although the microsphere deposition to non-target vessels is high for the Surefire and Endobar catheters, it can be seen that targeting is markedly improved when results for the manual and pressure-replicated injections are omitted.

The mechanisms for minimizing microsphere deposition in the esophageal branch vary by catheter type. The end-hole catheter lacks any deployable mechanism for preventing proximal transport of the microspheres; however, strong arterial flow away from the catheter tip propels them away from the esophageal branch. The lack of microspheres in the esophageal branch with the Surefire and Endobar catheters is due to their deployable anti-reflux mechanisms (permeable sheath and balloon, respectively).

### Microsphere distributions

**Fig. 6:**
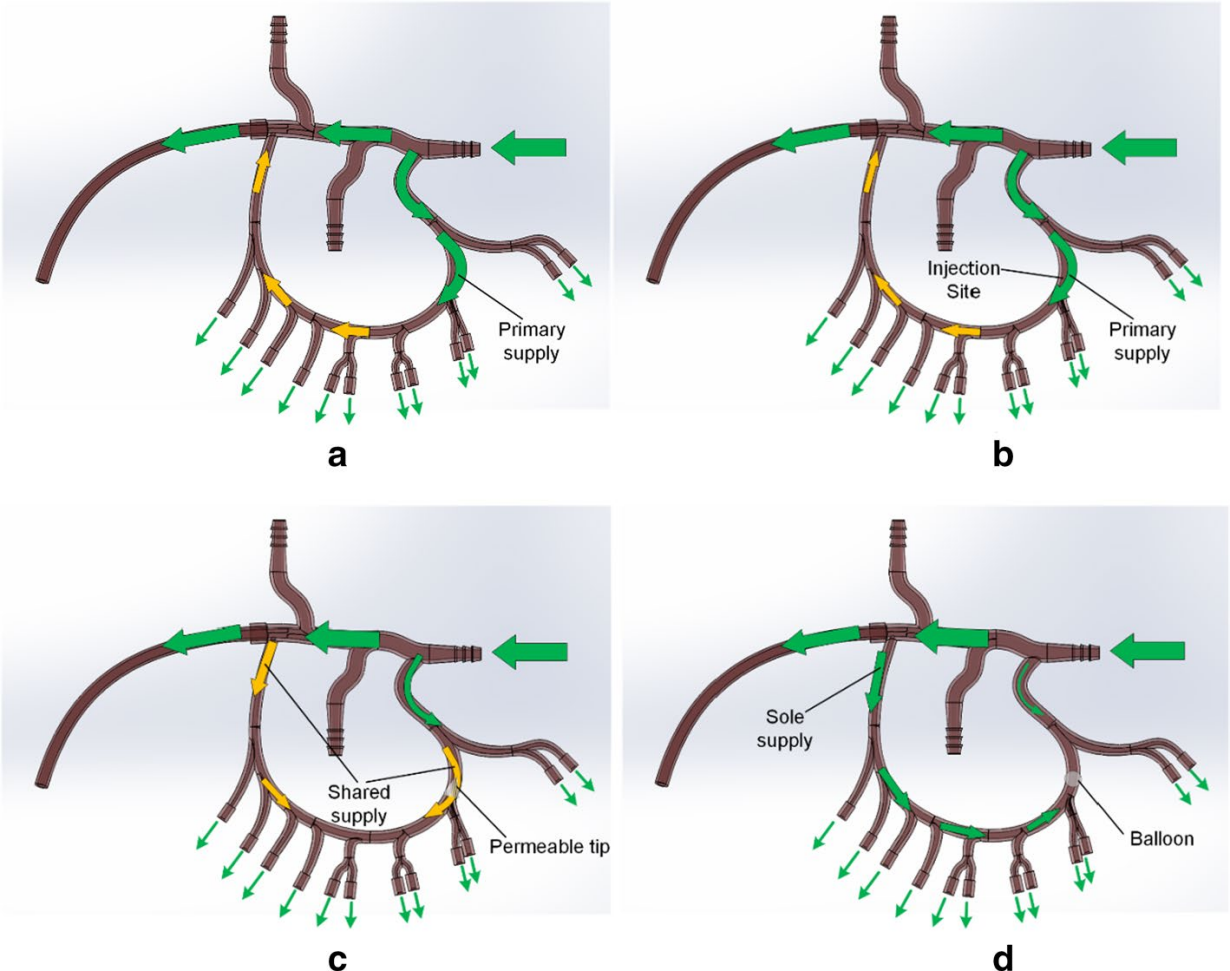
Observed (green) and hypothesized (yellow) pre-injection arterial flow vectors: **a** before the introduction of a catheter; **b** with an end-hole catheter (negligible flow obstruction), **c** with a Surefire catheter with deployed tip (partial flow obstruction at tip), and **d** with an Endobar catheter with inflated balloon (complete flow obstruction at balloon). Arrow sizes indicate relative volumetric flow rates

Results show a strong correlation between catheter type and microsphere penetration. With the end-hole catheter or in the absence of any catheter, arterial flow in the main collateral branch is predominantly from the left gastric artery (LGA) to the right gastric artery (RGA), promoting transport of microspheres toward the RGA and hepatic arterial branch (Fig. 6a, b). With the deployment of the Surefire tip or Endobar balloon, resistance to flow is introduced on the left side of the collateral branch. Consequently, arterial flow is predominantly from the RGA to the LGA (Fig. 6c, d). This right to left flow is most pronounced with the Endobar balloon, which almost entirely blocks the artery lumen. In these cases, arterial flow opposes the microsphere injection, limiting their penetration into the right gastric branches. Thus, the trend toward higher penetration from the Endobar to Surefire to end-hole catheters is intuitive. High-magnification video revealed propagation of the microspheres away from the catheter post-injection for the end-hole and Surefire catheters, indicating some left-to-right arterial flow; however, for Endobar injections, microspheres tended to oscillate within the same region or even back toward the catheter tip post-injection, indicating insignificant arterial flow just proximal to the catheter tip.

### Variability between replicate runs

Table 1 shows some variability between replicate runs. This behavior is a result of multiple factors, including radial catheter tip positioning, injection scheme, and viscoelastic behavior of the tubing. Unlike the Surefire and Endobar catheters, the end-hole catheter has no radial constraints within the vessel lumen; thus its position and orientation can vary significantly between runs, affecting microsphere depositions. Variability in outcomes for manual injections can be explained by significant differences in nominal injection rates (minimum 5.25 to maximum 10.80 mL/min). Pressure-replicated injection schemes have repeatable syringe movements but the undulating nature of the injection profile may promote turbulence at the injection site. Last, the constriction of the 1-mm clear tubes at the pinch valves is not entirely repeatable among tests, due to the viscoelastic nature of their PVC material. The tubes relax and thus constrict over time, necessitating adjustment of pinch valves between tests to achieve desired flow rates, i.e., identical pinch valve settings cannot be maintained for all tests.

## Conclusions

The presence of deployable mechanisms at the tip of a delivery catheter and the permeability of the mechanism had a significant effect on the distribution of embolic microspheres in a benchtop gastric arterial model. The Endobar catheter, featuring an impermeable balloon at its tip, yielded the highest vessel targeting accuracy (most proximal microsphere distributions) of the three catheter tests. In these tests, the injection method effect was not statistically significant across all catheter types and when considering the Endobar catheter/Endobar manifold combination vs. Endobar catheter injections with manual and pressure-replicated methods. However, the data trends and run-to-run variabilities suggest that with more replicates, and more statistical power, these injection method effects might be detectable.

## Materials and methods

### Arterial model

The rigid planar arterial model, fabricated using stereolithography processes, included left and right gastric, proper hepatic, gastroduodenal, and splenic arteries with flow terminating into 13 compliant distal vessels (Fig. 2). Central to the design of this model was replicating the hemodynamics (pressures, flow rates, pulsatile flow characteristics) and anatomical geometry (vessel diameters) both proximal and distal to the embolic injection point. For bariatric embolization procedures, embolic microspheres are infused from a delivery catheter at the left gastric artery, where they proceed toward the right gastric artery and small arterial branches supplying the lesser curvature of the stomach, ideally implanting in the latter. Flow rates, vessel diameters, and pressures vary substantially from the injection point to the target location. The model geometry was based on 3D CT imaging and anatomical measurements of vessel diameters. In specific, the left and right gastric arteries formed a collateral branch with two esophageal and ten gastric vessels (1.0 mm ID, 2.0 mm OD clear PVC tubing, labeled 1–12); the proper hepatic artery comprised a single branch (4.1 mm ID tubing, labeled 13). A pressure sensor was placed in the gastroduodenal artery for systemic pressure measurements, and pinch valves (located at each of the distal vessels) were included to regulate local pressures and flow rates. For microsphere collection and subsequent quantification, flow from each of these vessels was routed through laser-cut nylon mesh filters (100 µm filter rating) adhered to the base of nominal ½ inch PVC pipe couplings (Fig. 2b). Custom 3D-printed ABS brackets suspended the surgical tubing above the filter assemblies to visualize flow and verify embolization for each vessel (Fig. 2b).

**Fig. 7:**
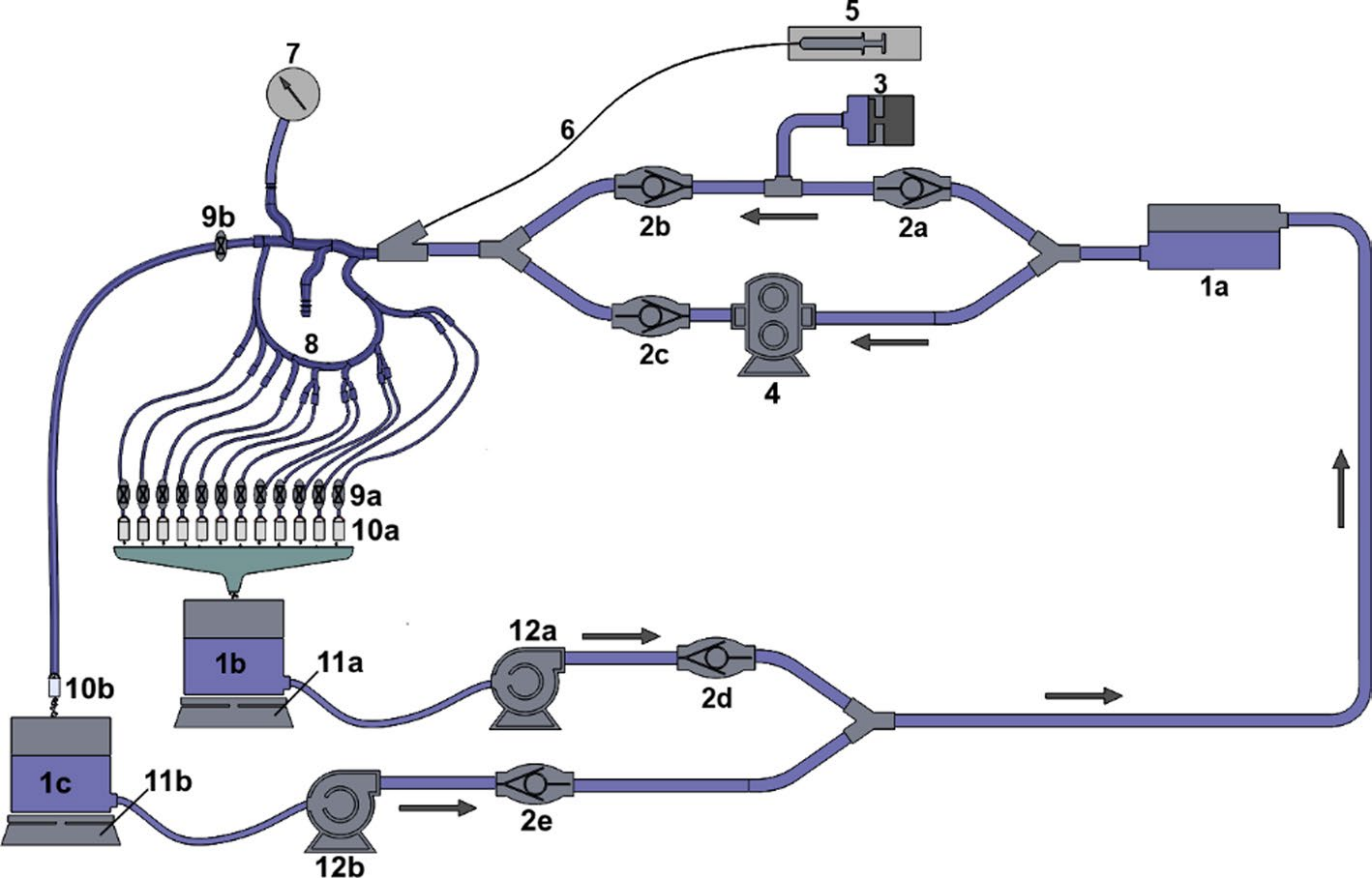
Arterial model schematic: (1a–1c) reservoirs, (2a–2e) one-way valves, (3) pulsatile pump, (4) gear pump, (5) syringe, (6) delivery catheter, (7) pressure transducer, (8) rigid arterial model, (9a, 9b) pinch valves, (10a, 10b) filters, (11a, 11b) laboratory scales, (12a, 12b) centrifugal pumps

A closed-loop, dynamically pressurized surrogate arterial system was assembled to facilitate experimental testing (Fig. 7). A custom-fabricated positive displacement pump and gear pump (components 3 and 4 in Fig. 7), connected in parallel, provided pulsatile flow with pressure profiles replicating those measured clinically (varying from 58 to 134 mmHg). The gear pump (Greylor Corporation, Cape Coral, FL) maintained the steady-state (time-invariant) component of the pressure waveform, while the positive displacement pump provided the pulsatile (time varying) component. Pinch valves at each of the 13 distal vessels allowed for precise adjustment of fluid resistance at each vessel. Real-time mass measurements of flow through the filters permitted the calculation of flow rates to both the proper hepatic artery and left and right gastric branches. Centrifugal pumps (components 12a and 12b in Fig. 7) were connected to the collection reservoirs to intermittently recirculate fluid back to the supply reservoir. One-way valves at the pumps prevented fluid leakage from the collection reservoirs between intermittent pump operations, ensuring accurate flow rate measurements. Flow through the proper hepatic artery was based on published right hepatic flow measurements [9–13] and the fraction of flow received by the right hepatic artery (RHA), 0.60 [12], yielding hepatic artery (HA) flow ranges from 48.3 to 375.0 mL/min. From this range, an experimental flow rate of 160 mL/min was chosen. A separate literature review was needed to determine physiological flow rates for the left and right gastric arteries [14–18]. Due to the lack of clinical measurements for right and left gastric arterial flow in humans, published arterial flow rate data for dogs were used to determine a ratio between left gastric and proper hepatic arterial flow (Table 3). The target arterial flow rate value (160 mL/min for the nominal case) was then divided by this ratio (2.84) to determine total flow to the gastric branches (56.3 mL/min).

**Table 3:** Published left gastric and hepatic arterial flow rates for dogs

Artery	Investigator(s)	Average flow (mL/min)	Average dog weight (kg)	Average flow per dog mass (mL/min/kg)	Average for anatomy (mL/min/kg)	Ratio, HA/LGA
Left gastric	Jacobson	39.0	14.0	2.79	2.70	2.84
	Naruse	33.9	13.0	2.61		
Hepatic	Burton-Opitz	143.5^a^	19.1^b^	7.8^a^ (7.5 by authors’ calculation)	7.65	
	Grodins et al.	144.0^a^	19.1^c^	7.5^a^		

^a^Reported in [16], ^b^from [17], ^c^from [18]

Since flow through the LGA is known to be significantly higher than that of the RGA, and since clinical RGA flow rates are not readily available in the literature, only LGA flow was considered in this study. Flow per branch through each of the esophageal branches was set equal to the average flow per gastric branch (5.6 mL/min). Because whole blood exhibits non-Newtonian characteristics at physiological flow rates, its viscosity varies widely (by nearly an order of magnitude) throughout the cardiac cycle [19, 20]. A study by Lowe et al. reported a mean blood viscosity (corrected for hematocrit level) of 3.49 cP at systolic shear rates (measured ex vivo) [21]. To achieve mean blood viscosity (corrected for hematocrit level) [21], a 25/75 glycerin/water solution was selected as the model’s working fluid. Measurements taken using a HAAKE™ Viscotester™ 550 (ThermoFisher Scientific, Waltham, MA) confirmed a viscosity of 3.48 ± 0.42 cP at a shear rate of 150 s-1.

### Microsphere Injection

The four gastric vessels immediately distal to the injection site (labeled 3 through 6 in Fig. 2) were targeted for embolization. Each run included an initial injection of 1.5 mL of a BeadBlock (BTG International Ltd., London, UK) mixture prepared by diluting BeadBlock solution (300–500 µm diameter for all tests of this study) from the manufacturer’s syringe with radiographic contrast and saline. Contrast was added at a 1:1 contrast:BeadBlock ratio and saline at a 2:1 saline:BeadBlock ratio. Each injection was followed by a saline flush, also of 1.5 mL volume. Preliminary test runs indicated that a dosage of 1.5 mL with the 3:1 dilution ratio provided an adequate number of microspheres to embolize the target vessels. This volume also matched the nominal pre-programmed dosage of the Endobar manifold.

Tests were repeated using three catheters: a standard end-hole micro-catheter (Renegade HI-FLO 2.8 Fr, Boston Scientific, Marlborough, MA), a Surefire anti-reflux catheter (SHF-38120-mT, TriSalus Life Sciences, Westminster, CO, formerly Surefire Medical), and an Endobar occlusion balloon catheter (Endobar Solutions, Orangeburg, NY). The latter is a prototype device, designed specifically for bariatric embolization. As of this publication, it has undergone clinical trials and is in the process of FDA approval. While the standard catheter has no anti-reflux features, the Surefire catheter includes an expandable semi-permeable tip; the Endobar catheter features a deployable balloon just proximal to its tip (Fig. 1).

For all three catheter types, manual injections, computer-controlled injections with a constant rate of 1.5 mL/min, and computer-controlled versions with a pre-programmed injection profile were performed. For the Endobar catheter, a fourth injection method was employed: a custom manifold developed specifically for LGA embolization with the Endobar catheter. Thus, the design consisted of a 3 × 3 + 1 statistical layout. All manual injections were performed by a single investigator (SJ) using a 6-mL syringe (Merit Medallion, Merit Medical, South Jordan, UT) and estimating dosage based on visual observation of the graduated marks. Due to their nature, the manual injections varied in rate. Post-test analysis of video found these to range from 5.25 to 10.80 mL/min.

**Fig. 8:**
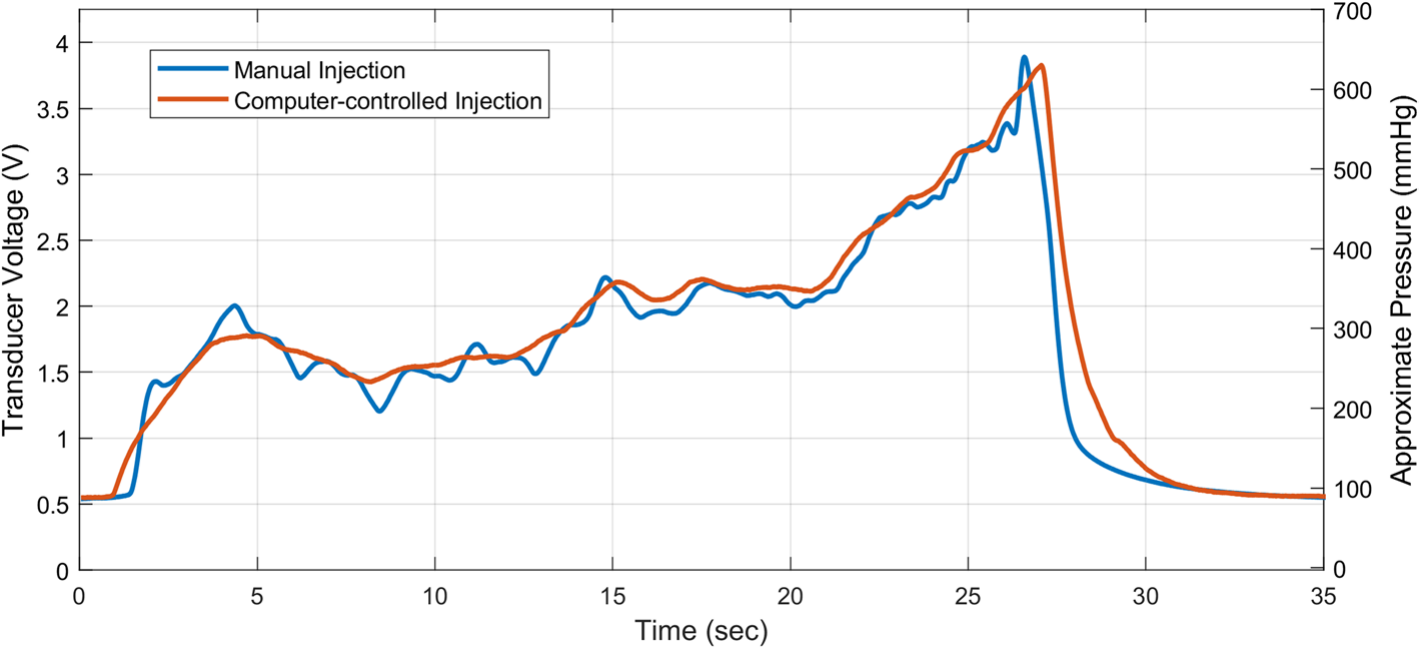
Manual and computer-controlled injection profiles: manual injection of BeadBlock solution by a clinician (blue) and subsequent profile produced by a computer-controlled syringe pump (red). Syringe pump displacement was subsequently scaled to produce 1.5 mL dosages. An offset in the pressure measurements (~ 97 mmHg) can be seen as a result of steady pressurization of the model

Computer-controlled injections (constant rate and pre-programmed injection profile) were performed with a custom-made syringe pump operated through a LabView interface; manual agitation of the delivery syringe, immediately before injection, was required to promote suspension of the particles. The pre-programmed injection profile was devised to replicate manual delivery by a clinician. Prior to these studies, pressure measurements were dynamically recorded at the proximal end of the catheter during manual delivery of 1.5 mL of BeadBlock solution into the statically pressurized arterial model (Fig. 8, blue profile). A clinician performed the injection through an end-hole catheter. An injection profile (displacement of the syringe plunger over time) was then devised (Fig. 8, red profile), through an iterative process, to replicate the injection pressure profile. Finally, syringe displacements were scaled within the profile to provide 1.5 mL dosages, in accordance with other delivery methods.

The custom manifold, used exclusively for the Endobar catheter, included an internal syringe and automated valves for de-airing the infusion lines, agitating the embolic solution (for particle suspension), and delivering the embolic solution at a pre-programmed dosage and injection rate. While designed to deliver a 1.5 mL dosage at 1.5 mL/min, measurements found the average dosage and injection rates to be 1.22 mL and 1.22 mL/min, respectively, for the conditions of this study. To deliver an equivalent volume of BeadBlock solution, the ratio of BeadBlock was increased with respect to contrast and saline (volume ratios for manifold injections: 30.5% BeadBlock solution, 23.2% contrast, 46.3% saline; ratio for all other injections: 25% BeadBlock solution, 25% contrast, 50% saline).

### Microsphere quantification

**Fig. 9:**
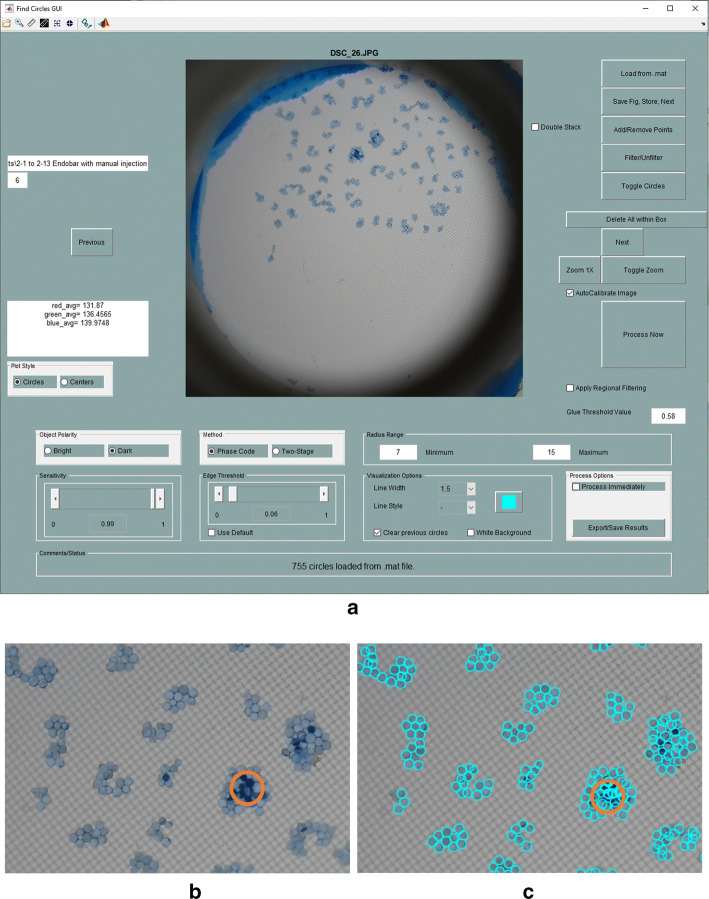
Quantification of microspheres with MATLAB-based image analysis: **a** GUI, **b** enlarged view of top center region of original image, and **c** detection of microspheres in region shown in **b**. Circled region denotes microspheres arranged in multiple layers

Following injection procedures, each filter was allowed to air dry for increased microsphere opacity. Photographs of each filter were captured using a DSLR camera (Nikon D500 with 85 mm AFS Micro NIKKOR lens, Nikon Corporation, Tokyo, Japan). Filters were backlit with the use of LED lights and an optical diffuser. A custom MATLAB application, developed through extensive adaptations of an open-source application (“FindCirclesGUI” by Brett Shoelson [22]), was used to automate the counting of microspheres in each image (Fig. 9). While most microspheres deposited on a single layer, microspheres depositing in multiple layers were distinguished by their darker color in relation to adjacent microspheres. In these cases, quantities of visible microspheres were doubled to account for stacking.

## Data Availability

The authors will make all data detailed in this paper freely available.

## Abbreviations

BMI: Body mass index
HA: Hepatic artery
LGA: Left gastric artery
LHA: Left hepatic artery
RGA: Right gastric artery

## References

[1] Anton K, Rahman T, Bhanushali A, Patel AA (2016). Bariatric left gastric artery embolization for the treatment of obesity: a review of gut hormone involvement in energy homeostasis. AJR Am J Roentgenol.

[2] Kipshidze N, Archvadze A, Bertog S, Leon MB, Sievert H (2015). Endovascular bariatrics: first in humans study of gastric artery embolization for weight loss. JACC Cardiovasc Interv..

[3] Gunn AJ, Oklu R (2014). A preliminary observation of weight loss following left gastric artery embolization in humans. J Obes..

[4] Hafezi-Nejad N, Bailey CR, Weiss CR (2020). Bariatric embolization: a narrative review of clinical data from human trials. Tech Vasc Interv Radiol.

[5] Weiss CR, Akinwande O, Paudel K, Cheskin LJ, Holly B, Hong K, Fischman AM, Patel RS, Shin EJ, Steele KE, Moran TH, Kaiser K, Park A, Shade DM, Kraitchman DL, Arepally A (2017). Clinical safety of bariatric arterial embolization: preliminary results of the BEAT obesity trial. Radiology.

[6] Bai Z, Qin Y, Deng G, Zhao G, Zhong B, Teng G (2018). Bariatric embolization of the left gastric arteries for the treatment of obesity: 9-month data in 5 patients. Obes Surg.

[7] Elens S, Roger T, Elens M, Rommens J, Sarafidis A, Capelluto E, Delcour C (2018). Gastric embolization as treatment for overweight patients; efficacy and safety. Cardiovasc Intervent Radiol.

[8] SAS Institute (2013). SAS/STAT 13.1 User’s Guide. Cary, NC: SAS Institute, Inc.

[9] Hübner GH, Steudel N, Kleber G, Behrmann C, Lotterer E, Fleig WE (2000). Hepatic arterial blood flow velocities: assessment by transcutaneous and intravascular Doppler sonography. J Hepatol.

[10] Hirata M, Akbar SM, Horiike N, Onji M (2001). Noninvasive diagnosis of the degree of hepatic fibrosis using ultrasonography in patients with chronic liver disease due to hepatitis C virus. Eur J Clin Invest.

[11] Han SH, Rice S, Cohen SM, Reynolds TB, Fong TL (2002). Duplex doppler ultrasound of the hepatic artery in patients with acute alcoholic hepatitis. J Clin Gastroenterol.

[12] Basciano CA. Computational particle hemodynamics analyses with applications to abdominal aortic aneurysms and liver targeting [PhD thesis], Raleigh, NC: North Carolina State University; 2010.

[13] Basciano CA, Kleinstreuer C, Kennedy AS, Dezarn WA, Childress E (2010). Computer modeling of controlled microsphere release and targeting in a representative hepatic artery system. Ann Biomed Eng.

[14] Jacobson ED, Dooley ES, Scott JB, Frohlich ED (1963). Effects of endotoxin on the hemodynamics of the stomach. J Clin Invest.

[15] Naruse S, Takagi T, Kato M, Ozaki T (1992). Interdigestive gastric blood flow: the relation to motor and secretory activities in conscious dogs. Exp Physiol.

[16] Werner AY, Horvath SM (1952). Measurement of hepatic blood flow in the dog by the bromsulphalein method. J Clin Invest.

[17] Burton-Opitz R (1910). The Vascularity of the liver. I. The flow of the blood in the hepatic artery. Q J Exp Physiol.

[18] Grodins FS, Osborne SL, Ivy AC, Goldman L (1941). The effect of bile acids on hepatic blood flow. Am J Physiol.

[19] Fung YC, Fung YC (1993). The Flow Properties of Blood. 1993 biomechanics: mechanical properties of living tissues.

[20] Baskurt OK, Meiselman HJ (2003). Blood rheology and hemodynamics. Semin Thromb Hemost.

[21] Lowe GD, Fowkes FG, Dawes J, Donnan PT, Lennie SE, Housley E (1993). Blood viscosity, fibrinogen, and activation of coagulation and leukocytes in peripheral arterial disease and the normal population in the Edinburgh Artery Study. Circulation.

[22] Shoelson. FindCirclesGUI. MathWorks File Exchange. 2011. https://www.mathworks.com/matlabcentraml/mlc-downloads/downloads/submissions/58686/versions/1/previews/FindCirclesGUI.m/index.html. Accessed on 3 Mar 2020.

